# High-sensitivity C-reactive protein to detect metabolic syndrome in a centrally obese population: a cross-sectional analysis

**DOI:** 10.1186/1475-2840-11-25

**Published:** 2012-03-14

**Authors:** Corine den Engelsen, Paula S Koekkoek, Kees J Gorter, Maureen van den Donk, Philippe L Salomé, Guy E Rutten

**Affiliations:** 1 Julius Center for Health Sciences and Primary Care, University Medical Center Utrecht, Utrecht, The Netherlands; 2 Huisartsenzorg IJsselstein, Locatie't Steyn, IJsselstein, The Netherlands

**Keywords:** Abdominal obesity, Metabolic syndrome, Screening, High-sensitivity C-reactive protein

## Abstract

**Background:**

People with central obesity have an increased risk for developing the metabolic syndrome, type 2 diabetes and cardiovascular disease. However, a substantial part of obese individuals have no other cardiovascular risk factors, besides their obesity. High sensitivity C-reactive protein (hs-CRP), a marker of systemic inflammation and a predictor of type 2 diabetes and cardiovascular disease, is associated with the metabolic syndrome and its separate components. We evaluated the use of hs-CRP to discriminate between centrally obese people with and without the metabolic syndrome.

**Methods:**

1165 people with central obesity but without any previous diagnosis of hypertension, dyslipidemia, diabetes or cardiovascular disease, aged 20-70 years, underwent a physical examination and laboratory assays to determine the presence of the metabolic syndrome (NCEP ATP III criteria). Multivariable linear regression analyses were performed to assess which metabolic syndrome components were independently associated with hs-CRP. A ROC curve was drawn and the area under the curve was calculated to evaluate whether hs-CRP was capable to predict the presence of the metabolic syndrome.

**Results:**

Median hs-CRP levels were significantly higher in individuals with central obesity with the metabolic syndrome (n = 417; 35.8%) compared to individuals with central obesity without the metabolic syndrome (2.2 mg/L (IQR 1.2-4.0) versus 1.7 mg/L (IQR 1.0-3.4); *p *< 0.001). Median hs-CRP levels increased with an increasing number of metabolic syndrome components present. In multivariable linear regression analyses, waist circumference and triglycerides were the only components that were independently associated with hs-CRP after adjusting for smoking, gender, alcohol consumption and the other metabolic syndrome components. The area under the ROC curve was 0.57 (95%-CI 0.53-0.60).

**Conclusions:**

Hs-CRP has limited capacity to predict the presence of the metabolic syndrome in a population with central obesity.

## Background

The metabolic syndrome (MetS) is a cluster of cardiovascular risk factors associated with an increased risk for developing cardiovascular disease and type 2 diabetes [1]. In recent years systemic inflammation - which can be measured by high sensitivity C-reactive protein (hs-CRP) - has become an important marker for cardiovascular disease and type 2 diabetes [2-4]. Moreover hs-CRP is associated with the MetS and its separate components. Early diagnosis of the MetS is desirable as lifestyle interventions and adequate treatment of risk factors associated with the MetS can prevent cardiovascular disease [5,6]. However, it is not clear whether hs-CRP can also predict the *presence *of the MetS. Previous research showed that measuring an increased waist circumference was a reliable first step in detecting individuals with the MetS and was easy to perform [7]. However, a substantial part of the individuals with central obesity have no other cardiovascular risk factors [8,9]. Further assessment of risk for cardiovascular disease, after initial (self-)measurement of waist circumference, involves physical examination and laboratory assays. An elevated hs-CRP could be used to further distinguish those with the MetS from those without and diminish the group qualifying for further examinations. Therefore the aim of this study was to evaluate the use of hs-CRP to discriminate those with the MetS from those without the MetS in a population with central obesity.

## Methods

### Study design and participants

A cross-sectional screening study was performed in five primary health care centres in IJsselstein, a small city in the centre of the Netherlands. The aim was to determine the feasibility of screening for the MetS by measuring waist circumference as a first step and to assess the prevalence of the MetS [7]. Almost 12000 individuals received a tape measure to determine their own waist circumference. They were 20 to 70 years old and had no previous diagnosis of cardiovascular disease, diabetes, hypertension or dyslipidemia, nor did they use medication for any of these conditions. Individuals with an increased self-measured waist circumference (≥ 88 cm in women; ≥ 102 cm in men) were invited for further examinations. Between September 2006 and May 2007, 1721 individuals with an increased self-measured waist circumference underwent all study procedures. Only participants with actual central obesity (a waist circumference ≥ 88 cm in women and ≥ 102 cm in men, measured by the investigators) were included in the present analyses.

The study was approved by the medical ethics committee of the University Medical Center Utrecht, the Netherlands. Written informed consent was obtained from all participants.

### Measurements

In a physical examination body weight, height, waist circumference and blood pressure were measured. Examinations were described in detail previously [7]. Venous blood samples were drawn after an overnight fast to determine fasting blood glucose, lipids (triglycerides, total cholesterol, LDL cholesterol and HDL cholesterol) and hs-CRP. LDL cholesterol was calculated using the Friedewald formula. Hs-CRP was analysed by latex-enhanced turbidimetric assay (Cobas Integra 800 Analyzer, Roche Diagnostics).

The participants completed a questionnaire to determine ethnicity, lifestyle factors (smoking habits, alcohol use, and physical activity), relevant medical history, socio-economic and demographic variables. Physical activity was assessed using the validated SQUASH questionnaire [10], which measures habitual activities with respect to occupation, leisure time, household tasks, transportation means, and other daily activities. The results were dichotomised based on the Dutch Standard Healthy Movement: a minimum of thirty minutes of exercise at least five days a week [11]. The use of alcohol was divided into three categories: no alcohol consumption, moderate alcohol consumption (one to 14 units per week for women, one to 21 units per week for men) and excessive alcohol consumption (more than 14 units per week for women, more than 21 units per week for men). Smoking was regarded positive when the participant was currently smoking tobacco; in case of former or never smoking it was regarded negative.

Participants were informed about the results of physical and laboratory examinations and in case of detected cardiovascular risk factors they received usual care by their general practitioner [12].

### Outcome measure

The presence of the MetS was defined by the criteria of the NCEP ATP III [13]. The diagnosis was made when at least three of the five following criteria were present: waist circumference ≥ 88 cm (women) or ≥ 102 cm (men); triglycerides ≥ 1.7 mmol/L; HDL cholesterol < 1.3 mmol/L (women) or < 1.0 mmol/L (men); blood pressure ≥ 130/≥ 85 mmHg; fasting glucose ≥ 6.1 mmol/L.

### Data analyses

All statistical analyses were performed with SPSS, version 15.0 (SPSS, Chigaco, Illinois). Categorical variables are reported as numbers and percentages, continuous variables as means with standard deviations (SD) and non normally distributed variables as median with interquartile range (IQR). Differences between groups were analysed with Chi-square tests for categorical variables, independent t-tests for normally distributed continuous variables and Mann-Whitney tests for non normally distributed continuous variables. Hs-CRP was divided into three categories based on the cut-off points for risk stratification for cardiovascular risk [14]. A level below 1.0 mg/L is considered a low cardiovascular risk, between 1.0 and 3.0 mg/L an intermediate risk and between 3.0 and 10.0 mg/L a high risk. A level above 10.0 mg/L is associated with active infection and therefore not applicable in the risk estimation [15]. Individuals with an hs-CRP level above 10.0 mg/L were excluded from the analyses.

Univariable linear regression analyses were used to calculate the p for trend over hs-CRP categories, in which the hs-CRP categories were used as independent variable. A multivariable linear regression analysis was performed to assess which MetS components were independently associated with hs-CRP. In this analysis hs-CRP and triglycerides were logtransformed because of their skewed distribution. Waist circumference and HDL cholesterol were also transformed, taking into account the gender specific thresholds. The gender specific threshold was extracted from the values obtained in the examinations. The new variables indicate the absolute difference with the gender specific threshold. A ROC curve was drawn and the area under the curve was calculated to evaluate whether hs-CRP is capable to predict the presence of the MetS. Sensitivity, specificity and the positive and negative predictive values for different hs-CRP cut-off points were calculated.

## Results

Of the 1721 participants with a self-measured increased waist-circumference that completed all study procedures 108 individuals were excluded from the analyses because of an hs-CRP above 10.0 mg/L. 1165 individuals had an actual increased waist circumference, measured by the investigator. The mean age in the participants with an actual increased waist circumference was 48.4 (SD 10.7) years, 92.7% were Caucasian and 65.2% were female. The prevalence of MetS in this population with central obesity was 35.8% (n = 417). Table 1 shows the patient characteristics for the individuals with an increased waist circumference with and without the MetS. The median hs-CRP for the total population was 1.9 mg/L (IQR 1.1-3.6). In the population with the MetS the median hs-CRP was 2.2 mg/L (IQR 1.2-4.0), compared to 1.7 mg/L (IQR 1.0-3.4) in the group without the MetS (*p *< 0.001).

**Table 1:** Patient characteristics for the total population and according to the presence of the metabolic syndrome

Characteristics	Total n = 1165	Metabolic syndrome present n = 417	Metabolic syndrome absent n = 748	P-value
Age (years)	48.4 ± 10.7	48.5 ± 10.5	48.3 ± 10.8	0.76
Gender (%, female)	65.2	48.0	74.9	< 0.001
Waist circumference (cm)				
- Female	97.7 ± 8.1	100.3 ± 9.2	96.7 ± 7.4	< 0.001
- Male	109.7 ± 6.6	110.5 ± 6.9	108.9 ± 6.3	0.01
BMI (kg/m^2^)	29.5 ± 3.6	30.3 ± 3.6	29.1 ± 3.5	< 0.001
Blood pressure (mmHg)				
- Systolic	137.7 ± 17.3	144.8 ± 15.8	133.7 ± 16.8	< 0.001
- Diastolic	84.8 ± 9.3	88.7 ± 7.9	82.7 ± 9.3	< 0.001
Triglycerides (mmol/L)	1.2 (0.9-1.8)	1.9 (1.7-2.4)	1.0 (0.8-1.3)	< 0.001
HDL cholesterol (mmol/L)				
- Female	1.6 ± 0.4	1.3 ± 0.3	1.7 ± 0.4	< 0.001
- Male	1.2 ± 0.3	1.1 ± 0.3	1.4 ± 0.2	< 0.001
LDL cholesterol (mmol/L)	3.2 ± 0.9	3.4 ± 0.9	3.2 ± 0.8	< 0.001
Fasting glucose (mmol/L)	5.0 ± 0.9	5.4 ± 1.4	4.9 ± 0.5	< 0.001
Hs-CRP (mg/L)	1.9 (1.1-3.6)	2.2 (1.2-4.0)	1.7 (1.0-3.4)	< 0.001
Physical activity meeting Dutch	59.5	56.1	61.3	0.09
Standard Healthy Movement^‡ ^(%)				
Current smoking (%)	20.3	24.6	17.9	0.01
Alcohol consumption (%)				0.03
- None	28.3	31.1	26.7	
- Moderate	64.5	59.8	67.1	
- Excessive	7.2	9.2	6.2	

Data are reported as means ± standard deviation or percentage. Non normally distributed variables are reported as median (25th-75th percentile).

^‡ ^Dutch Standard Healthy Movement: a minimum of thirty minutes of exercise at least five days a week.

With an increasing number of MetS components present, median hs-CRP increased from 1.5 mg/L (IQR 0.9-2.9) to 1.9 (IQR 1.1-3.5), 2.1 (IQR 1.1-4.0), 2.4 (IQR 1.5-4.5) and 2.2 mg/L (IQR 1.4-4.4) in the groups with respectively one (n = 234), two (n = 514), three (n = 301), four (n = 103) and five (n = 13) components (*p *for trend < 0.001).

Table 2 shows the clinical and biochemical characteristics according to hs-CRP categories. A significant linear trend over increasing hs-CRP categories was seen for the presence of the MetS and for three of its components: waist circumference, triglycerides and HDL cholesterol. BMI, gender, current tobacco smoking and current alcohol consumption also showed a significant linear trend. In a multivariable linear regression analysis, waist circumference and triglycerides were the only MetS components that were independently associated with hs-CRP, after adjusting for smoking, gender, alcohol consumption and the other MetS components (Table 3).

**Table 2:** Patient characteristics across hs-CRP cardiovascular risk level categories

	hs-CRP (mg/L)			P for trend
	
	< 1.0 n = 254	≥ 1.0-≤ 3.0 n = 545	> 3.0-< 10.0 n = 366	
Age (years)	47.9 ± 10.6	49.6 ± 10.6	47.0 ± 10.7	0.15
Gender (%, female)	61.0	61.5	73.8	< 0.001
Waist circumference (cm)				
- Female	95.0 ± 5.5	96.8 ± 7.6	100.3 ± 9.0	< 0.001
- Male	106.9 ± 3.4	110.0 ± 6.2	112.1 ± 8.6	< 0.001
Triglycerides (mmol/L)	1.1 (0.8-1.6)	1.2 (0.9-1.8)	1.3 (0.9-1.8)	< 0.01
LDL cholesterol (mmol/L)	3.2 ± 0.9	3.2 ± 0.8	3.2 ± 0.9	0.78
HDL cholesterol (mmol/L)				
- Female	1.7 ± 0.4	1.6 ± 0.4	1.5 ± 0.4	< 0.001
- Male	1.3 ± 0.3	1.2 ± 0.3	1.2 ± 0.3	0.02
Blood pressure (mmHg)				
- Systolic	135.7 ± 15.4	138.2 ± 17.5	138.3 ± 18.1	0.09
- Diastolic	84.2 ± 8.5	84.8 ± 9.3	85.3 ± 9.8	0.12
Fasting glucose (mmol/L)	5.0 ± 0.7	5.0 ± 1.0	5.1 ± 1.0	0.10
BMI (kg/m^2^)	28.3 ± 3.0	29.4 ± 3.2	30.6 ± 4.2	< 0.001
Metabolic syndrome (%)	29.5	34.7	41.8	< 0.01
Current smoking (%)	19.3	17.6	25.0	0.05
Drinking alcohol (%)				< 0.01
- None	20.9	27.9	34.0	
- Moderate	70.5	64.9	59.7	
- Excessive	8.7	7.2	6.3	
Physical activity meeting Dutch Standard Healthy Movement^‡ ^(%)	55.5	61.3	59.5	0.41

Data are reported as means ± standard deviation or percentage. Non normally distributed variables are reported as median (25th-75th percentile).

^‡ ^Dutch Standard Healthy Movement: a minimum of thirty minutes of exercise at least five days a week.

**Table 3:** Associations between metabolic syndrome components and hs-CRP in multivariable linear regression analysis

	B	(95%-CI)	P-value
Waist circumference (cm)	0.03	(0.02; 0.03)	< 0.001
Triglycerides (mmol/L)	0.21	(0.10; 0.32)	< 0.001
HDL cholesterol (mmol/L)	-0.06	(-0.20; 0.09)	0.43
Systolic blood pressure (mmHg)	0.00	(-0.00; 0.01)	0.11
Fasting glucose (mmol/L)	-0.02	(-0.08; 0.03)	0.37

B, unstandardized regression coefficient, adjusted for gender, smoking status, alcohol consumption and the other metabolic syndrome components. CI, confidence interval.

**Figure 1:**
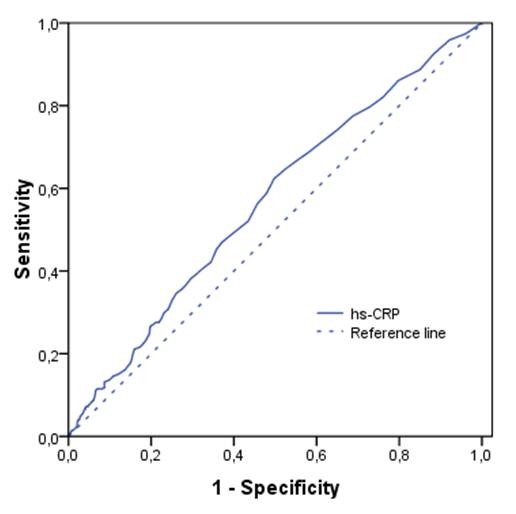
**ROC curve showing that the capacity of hs-CRP to detect the presence of the metabolic syndrome in a population with central obesity is limited**. The area under the ROC curve is 0.57 (95%-CI 0.53-0.60).

The area under the ROC curve for hs-CRP was 0.57 (95%-CI 0.53-0.60) (Figure 1).

At a cut-off point for hs-CRP at a level of 1.0 mg/L the sensitivity and specificity of hs-CRP for the MetS were 82% and 24%, respectively. The positive and negative predictive values for this level were 38% and 71% respectively. At a cut-off level of 3.0 mg/L the sensitivity and specificity were 37% and 72% respectively. The positive and negative predictive values for this level were 42% and 67% respectively.

## Discussion

Median hs-CRP levels were significantly higher in individuals with central obesity with the MetS compared to individuals with central obesity without the MetS. Median hs-CRP levels increased with an increasing number of MetS components present. The presence of the MetS, waist circumference, triglycerides and HDL cholesterol showed a significant trend over increasing hs-CRP categories. However the area under the ROC curve was 0.57, indicating that hs-CRP cannot predict the presence of the MetS in a population with central obesity.

Strengths of this study were the large number of people and the wide variety in age, also including younger people. Our study population consisted of individuals without known cardiovascular disease, diabetes, hypertension or dyslipidemia; besides an increased waist circumference they were considered healthy. However because of their increased waist circumference they were at risk for developing cardiovascular disease and were therefore a relevant population for studying the use of hs-CRP as a screening tool for *early *detection of the MetS.

A limitation of this study was the cross-sectional design. We only performed measurements at one time point. Hs-CRP is a sensitive marker for acute phase inflammation and has a high within-subject variability [16]. A value above five might indicate an increased cardiovascular risk, but could also be an hs-CRP returning to normal low levels after an infection [14]. However, since we intended to explore the predictive value of hs-CRP measured at one time point for detecting the presence of the MetS, our design reflects routine in daily practice.

We found hs-CRP levels to be higher in centrally obese individuals with the MetS, compared to those without. This is in line with several studies which found higher hs-CRP levels in individuals with the MetS, both in obese and non-obese populations and in Caucasian and non-Caucasian populations [17-25]. Also a linear increase in hs-CRP levels with an increasing number of components of the MetS was described previously [18-21,23,25-27]. Over increasing hs-CRP risk level categories the percentage of people with the MetS increased significantly. A similar significant trend was found for mean waist circumference and HDL cholesterol and median triglyceride levels. But only waist circumference and triglycerides showed a significant independent association with hs-CRP in multivariable analysis. Several studies have assessed the relation between hs-CRP and different MetS components. In univariable analyses most of them showed significant associations for the individual MetS components. Studies that also assessed multivariable associations, thereby adjusting for other MetS components, mostly report that central obesity was the major determinant of elevated hs-CRP levels in individuals with the MetS. The other MetS components do not, or only marginally, increase hs-CRP level [17-20,26,28]. This might explain why hs-CRP cannot be used to predict the presence of the MetS in our study, indicated by an area under the ROC curve of 0.57.

The relationship between central obesity and increased levels of hs-CRP has been well studied. Adipose tissue is known to secrete cytokines that stimulate the production of hs-CRP in the liver, but adipose tissue itself may also secrete hs-CRP and thereby raise hs-CRP levels [29]. Genetic polymorphisms could partially explain the inter-individual variability observed in the inflammatory profile of obese patients and the inter-individual variability in metabolic perturbations associated with obesity [30,31].

Although central obesity seems to be the major determinant of elevated hs-CRP levels in the MetS, significant independent associations between hs-CRP and other components were found. As in our study, Aronson et al. found an independent association between triglyceride level and hs-CRP. In addition they found associations between hs-CRP and glucose level and HDL cholesterol. However, this only accounted for ~1% of the variability in CRP levels [26].

Two other studies also found an independent association between hs-CRP and fasting glucose [21,32]. We did not find such an association, which might be due to the limited number of individuals with a high glucose level (≥ 6.1 mmol/L) in our study population (n = 66; 5.7%). General practitioners in the Netherlands are alert in screening for diabetes as a result of which the number of patients with undiagnosed diabetes is limited [33].

## Conclusions

In our population with central obesity, measurement of hs-CRP cannot be used to further discriminate MetS status. The degree of central obesity seemed to be the main determinant of an increased hs-CRP level; the association with other MetS components was not strong enough to enable further discrimination. While hs-CRP does not distinguish metabolic syndrome well on its own, it may have utility in conjunction with other tests, which requires further testing. In addition, hs-CRP could still be used as a prognostic marker of cardiovascular disease and diabetes. This would enable physicians to determine which centrally obese individuals should be encouraged most strongly to adapt a healthier lifestyle.

## Abbreviations

Hs-CRP: High-sensitivity C-reactive protein; MetS: Metabolic syndrome

## Competing interests

The authors declare that they have no competing interests.

## Authors' contributions

CdE and PSK researched the data, performed the statistical analyses and wrote the manuscript. KJG, MvdD, PLS and GER contributed to the discussion. All authors were involved in the design of the study and read and approved the final manuscript.
